# Influence of model boundary conditions on blood flow patterns in a patient specific stenotic right coronary artery

**DOI:** 10.1186/1475-925X-14-S1-S6

**Published:** 2015-01-09

**Authors:** Biyue Liu, Jie Zheng, Richard Bach, Dalin Tang

**Affiliations:** 1 Department of Mathematics, Monmouth University, West Long Branch, NJ 07764, USA; 2 Mallinckrodt Institute of Radiology, Washington University, St. Louis, MO, USA; 3 Cardiovascular Division, Washington University School of Medicine, Saint Louis, MO, USA; 4 School of Biological Science and Medical Engineering, Southeast University, Nanjing 210096, China; 5 Department of Mathematical Sciences, Worcester Polytechnic Institute, Worcester, MA 01609, USA

## Abstract

**Background:**

In literature, the effect of the inflow boundary condition was investigated by examining the impact of the waveform and the shape of the spatial profile of the inlet velocity on the cardiac hemodynamics. However, not much work has been reported on comparing the effect of the different combinations of the inlet/outlet boundary conditions on the quantification of the pressure field and flow distribution patterns in stenotic right coronary arteries.

**Method:**

Non-Newtonian models were used to simulate blood flow in a patient-specific stenotic right coronary artery and investigate the influence of different boundary conditions on the phasic variation and the spatial distribution patterns of blood flow. The 3D geometry of a diseased artery segment was reconstructed from a series of IVUS slices. Five different combinations of the inlet and the outlet boundary conditions were tested and compared.

**Results:**

The temporal distribution patterns and the magnitudes of the velocity, the wall shear stress (WSS), the pressure, the pressure drop (PD), and the spatial gradient of wall pressure (WPG) were different when boundary conditions were imposed using different pressure/velocity combinations at inlet/outlet. The maximum velocity magnitude in a cardiac cycle at the center of the inlet from models with imposed inlet pressure conditions was about 29% lower than that from models using fully developed inlet velocity data. Due to the fact that models with imposed pressure conditions led to blunt velocity profile, the maximum wall shear stress at inlet in a cardiac cycle from models with imposed inlet pressure conditions was about 29% higher than that from models with imposed inlet velocity boundary conditions. When the inlet boundary was imposed by a velocity waveform, the models with different outlet boundary conditions resulted in different temporal distribution patterns and magnitudes of the phasic variation of pressure. On the other hand, the type of different boundary conditions imposed at the inlet and the outlet did not have significant effect on the spatial distribution patterns of the PD, the WPG and the WSS on the lumen surface, regarding the locations of the maximum and the minimum of each quantity.

**Conclusions:**

The observations from this study indicated that the ways how pressure and velocity boundary conditions are imposed in computational models have considerable impact on flow velocity and shear stress predictions. Accuracy of *in vivo *measurements of blood pressure and velocity is of great importance for reliable model predictions.

## Introduction

The coronary arteries are a common and important site of atherosclerotic lesion development [1]. It is believed that mechanical stresses resulting from intravascular pressure and flow may contribute to the pathogenesis of atherosclerosis. The curvature and the presence of stenosis in coronary arteries result in considerable change to the flow field. Flow disturbances associated with a stenosis could contribute to modifications in the rate of plaque development, the direction of plaque extension and the composition of the plaque [2-4]. A detailed hemodynamic evaluation of disturbed flow and the spatial and temporal flow distribution patterns may give additional insight to understanding the progression of atherosclerosis and may have useful clinical value. However, it is difficult to measure local flow patterns and mechanical forces *in vivo *with sufficient accuracy. Computational models become a useful tool in that regard.

In recent decades, much progress had been made in research coupling medical imaging and computational fluid dynamics (CFD) to study cardiovascular hemodynamics [1,4-9]. The methods developed could provide investigators with powerful new tools, rivaling and even surpassing experimental fluid mechanics methods to investigate the mechanisms of disease and to design medical devices and therapeutic interventions [8]. Torii et al. investigated the effects of wall compliance on coronary hemodynamics by coupling FSI analysis of the human RCA in conjunction with physiological velocity and pressure waveforms [4]. Johnston et al. compared models of Newtonian and non-Newtonian flow in four healthy right coronary arteries reconstructed from biplane angiograms [5]. Zeng et al. examined the effects of cardiac motion on right coronary artery hemodynamics [9]. Myers et al. investigated the effects of arterial geometry, pulsatile flow condition and dynamic vessel motion on flow patterns in a model with a patient specific right coronary artery geometry [1]. Tang et al. provided a review of the key steps and findings of their group's in image-based models for human carotid and coronary plagues [6].

In general the solution of a system of partial differential equations is only uniquely determined if the boundaries of the domain are imposed with necessary conditions. Therefore, to perform the 3D simulations of blood flow in human arteries, proper boundary conditions need to be specified at the inlet and the outlet boundaries when solving the governing Navier-Stokes equations. In addition to the consideration of the well-posedness of the computational problem, more natural and realistic conditions relating pressure and velocity waveforms for image-based modeling may enable researchers to better quantify and predict the local pressure fields and flow distribution in the upstream and downstream domains of the patient specific stenotic artery. On the other hand, improperly imposed boundary conditions may result in misleading hemodynamic information. Usually the velocity profile or the pressure profile is explicitly prescribed at the inlet. There is a variety of approaches for the outlet boundary condition. Three of the commonly used simple outlet conditions are prescribed velocity or pressure profile, or zero normal traction. Different combinations of the above mentioned boundary conditions at the inlet and the outlet have been applied by many researches [4,5,9-15].

Several authors have studied the influence of the flow waveform and the spatial profile of the inlet velocity on CFD predictions of blood flow in human arteries [1,16-18]. Mark et al. demonstrated the importance of unsteadiness in a left anterior descending coronary artery flow phantom [17]. Ethier et al. examined the flow waveform effects on end-to-side anastomotic flow patterns [16]. Myers et al. investigated the effects of the inlet velocity profile and flow waveform on the flow patterns on the hemodynamics in the proximal, medial, and distal arterial regions of human right coronary artery. In their study, a zero-traction condition was imposed at the model outlet and a velocity profile was imposed at the inlet. They compared three different inlet velocity profiles: a fully developed inlet velocity profile, a "blunt" inlet velocity profile, and a fully developed Dean-type velocity profile. They also used two flow waveforms: sinusoidal and physiological waveforms in the study. They concluded that changes in the inlet velocity profiles did not produce significant changes in the arterial velocity and wall shear stress patterns and the waveform shape is not a significant factor in the right coronary time-averaged WSS features [1]. Morbiducci et al. studied the influence of assumptions regarding velocity profiles at the inlet section of the ascending aorta. They concluded that the plausibility of the assumption of idealized velocity profiles as inlet boundary conditions can lead to misleading representations of the aortic hemodynamics both in terms of disturbed shear and bulk flow structures [18]. However, not much work has been reported on comparing the effect of the different combinations of the inlet/outlet boundary conditions on the quantification of the pressure field and flow distribution patterns. The aim of this work is to investigate the influence of the different types of boundary conditions imposed at the inlet/outlet on the spatial and the temporal distribution patterns of the blood flow in a patient-specific stenotic right coronary artery.

## Patient-specific plaque geometry and flow data

**Figure 1:**
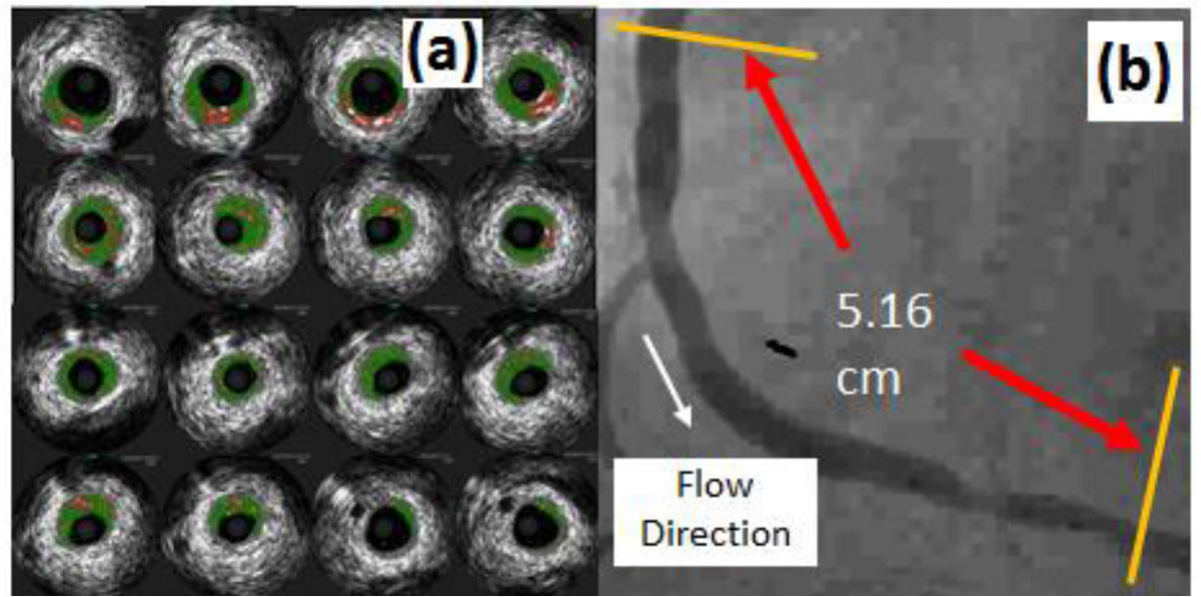
**(a) Selected IVUS slices from a 44-slice set, (b) Angiographic image of the coronary segment**.

**Figure 2:**
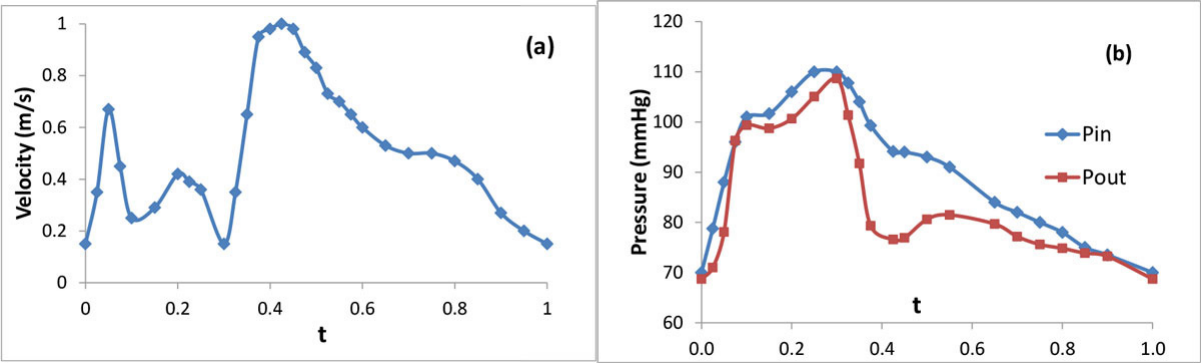
**Pulse waveforms of the velocity in (a) and pressure Pin in (b) extracted at the inlet of the artery segment from the patient**. Pout in (b) was calculated at the outlet of the artery.

**Figure 3:**
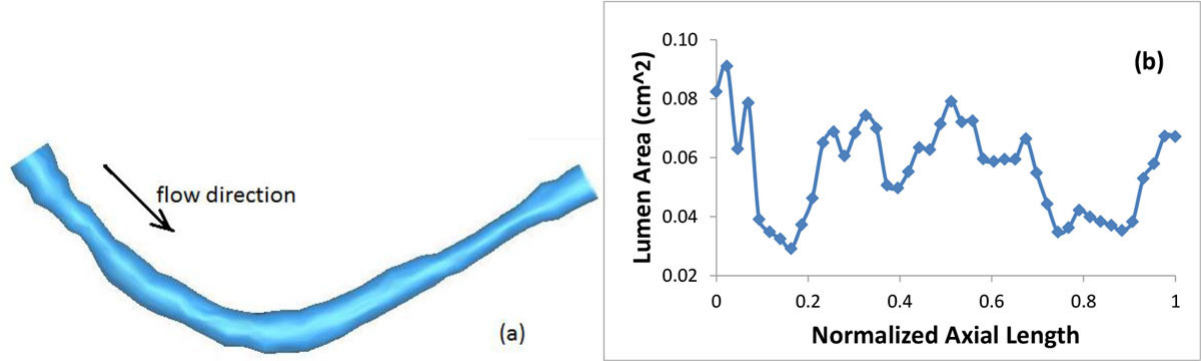
**(a) Geometry of the computational domain, (b) Lumen cross-section area**.

An image-based model of a stenotic right coronary artery was reconstructed based on the lumen contour curves extracted from a 44-slice *in vivo *3D IVUS dataset acquired from a patient, using a 20-MHz, 2.9-F phased-array Eagle Eye Gold IVUS catheter (Volcano Corporation, Rancho Cordova, CA), with patient consent obtained [19]. Figure 1(a) presents selected IVUS slices from the 44-slice dataset. Figure 1(b) is an enlarged angiographic image of the coronary segment with the flow direction and the length labeled. The velocity and pressure pulse waveforms at the inlet were extracted (see Figure 2(a) and Pin in Figure 2(b)) from on-site blood pressure and flow velocity data at the location of the first IVUS slice using a Combo-Wire XT 9500 (Volcano Therapeutics, Inc.) 0.014-inch guide-wire with a Doppler flow velocity sensor. A normalized time length t/t_p _= 1 was used for simplicity. Figure 3(a) shows the geometry of the stenotic right coronary artery segment constructed based on the lumen contours. Figure 3(b) is the plot of lumen cross-section areas from the inlet to the outlet of the artery segment with the horizontal axis as the normalized axial length. The lumen cross-section area plot indicates that it is a patient specific right coronary artery with multi-stenoses. The first stenosis near the inlet has a reduction of 66% approximately in lumen cross-section area and the second stenosis near the outlet has a reduction of 47% approximately in lumen cross-section area.

## The flow model and numerical method

### Flow model

The blood was assumed as a laminar, incompressible, non-Newtonian viscous fluid obeying the Carreau model with the viscosity-shear rate relation [20]:

(1) $$\eta =\eta_{\infty }+\left(\eta_{0}-\eta_{\infty }\right){\left[1+{\left(\lambda \dot{\gamma }\right)}^{2}\right]}^{\frac{n-1}{2}},$$

where *η*_0 _= 0.056 *Pa∙s *is the zero shear rate viscosity, *η*_∞ _= 0.00345 *Pa∙s *is the infinite shear rate viscosity, $\dot{\gamma }$ is the shear rate, *λ *= 3.313*s *is a parameter, and n = 0.3568 is a dimensionless parameter. In the computations, the blood density *ρ *was assumed to be constant at 1050 *kg*/*m^3^*.

The time dependent three dimensional Navier-Stokes equations were used as the governing equations. A no-slip condition was applied to the velocities at the lumen wall, treated to be inelastic and impermeable. To examine the influence of the boundary conditions on the pressure fields and the flow distribution of the stenotic right coronary artery, the following commonly applied five combinations of inlet/outlet boundary conditions were utilized in this study with experimental pressure and/or velocity data used when applicable:

(1) V-NS0 model: A fully developed velocity profile with pulse waveform (Figure 2(a)) was imposed at the inlet and a zero normal stress condition was imposed at the outlet.

(2) P-V model: A time dependent pressure with waveform Pin (Figure 2(b)) was imposed at the inlet. A fully developed velocity profile with pulse waveform (Figure 2(a)) was multiplied by a scalar based on the sizes of the inlet/outlet cross sections such that the flow rate at the outlet is equal to that at the inlet. Then this adjusted velocity waveform was imposed at the outlet.

(3) V-P model: A fully developed velocity profile with pulse waveform (Figure 2(a)) was imposed at the inlet and a time dependent pressure with waveform Pout (as shown in Figure 2(b)) was imposed at the outlet.

(4) P-P model: A time dependent pressure with waveform Pin and Pout (as shown in Figure 2(b)) was imposed at the inlet and the outlet boundary, respectively.

(5) P-NS0 model: A time dependent pressure with waveform Pin (Figure 2(b)) was imposed at the inlet and a zero normal stress condition was imposed at the outlet.

### Numerical method

Computer simulations were carried out using COMSOL 4.4. The inlet and the outlet of the artery were extended in length by .2 cm in the direction normal to the inlet and the outlet cross sections to reduce the influence of the boundary conditions in the region of interest. The finite element method over an unstructured tetrahedral mesh was adopted to solve the governing equations of the fluid motion into the right coronary artery. Four consecutive cardiac cycles were simulated to ensure that the flow was truly periodic. To confirm the independence of the numerical solutions on spatial mesh, computations were repeated over different sizes of mesh. The relative errors of the solutions between different meshes were less than 0.5%. The pressure drop along the artery length is defined as P-P_In_, where P_In _is a reference pressure chosen simultaneously as the blood pressure at the inlet of the coronary artery. The spatial gradient of the wall pressure is defined as

(2) $$WPG=\sqrt{{\left(\frac{\partial p}{\partial x}\right)}^{2}+{\left(\frac{\partial p}{\partial y}\right)}^{2}+{\left(\frac{\partial p}{\partial z}\right)}^{2}}.$$

In the process of the computer simulations using the models with different combinations of the inlet/outlet boundary conditions, the computation of the P-V model was first carried out. This model applied the real data measured from the patients at the boundaries and thus its simulation results were used for the comparison with other models. The blood pressure at the outlet boundary obtained from P-V model was averaged on the lumen cross section at each time during the cardiac cycle, resulting in a pressure waveform named Pout (see Pout in Figure 2(b)). This pulse pressure waveform was then used as the boundary condition at the outlet for the V-P model and the P-P model. In all models imposed with pressure boundary condition at either the inlet or the outlet the pressure is assumed as uniform spatially over the inlet or the outlet cross section.

### Validation of numerical solutions

**Figure 4:**
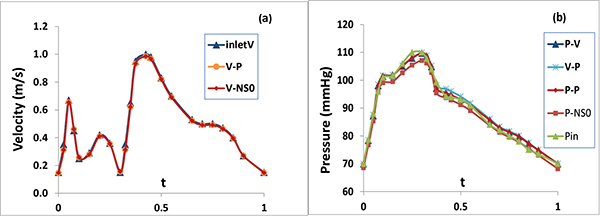
**Comparison of the simulated (a) velocity magnitude and (b) pressure at the center of the first lumen cross-section slice with acquired data from the patient (inletV and Pin)**.

Numerical solutions were validated by comparing the simulated blood velocity and pressure with the acquired on-site blood pressure and flow velocity data from the patient. The inletV in Figure 4(a) and the Pin in Figure 4(b) are the magnitude of the velocity pulse waveform and the pressure pulse waveform extracted from the patient, respectively. The computer simulations were performed over the computational domain with an extended inlet tube and an extended outlet tube. The inletV and the Pin were imposed at the entry of the extended inlet tube for V-P and V-NS0 models and for P-V, P-P and P-NS0 models, respectively. Figure 4(a) compared inletV with the computed magnitude of the velocity in a cardiac cycle at the center of the first lumen cross-section IVUS slice of the artery segment for V-NS0 and V-P models. The good matching of the three plots validates the computer simulations of the blood flow velocity. We can also see that as the imposed fully developed flow moves through the extended inlet tube it remains its magnitude and its fully developed profile when entering the first IVUS slice of right coronary artery segment. Figure 4(b) compared the Pin with the computed blood pressure in a cardiac cycle at the center of the first lumen cross-section IVUS slice of the artery segment for P-V, P-P, V-P and P-NS0 models. The plots of five curves also show a good matching. The maximum relative error of 3% for P-V, P-P and V-P models and 4% for P-NS0 model are mainly caused by the expected blood pressure drop/variation between the entry of the extended inlet tube and the first lumen IVUS slice of the right coronary artery segment. It is worth of mentioning the good matching between the Pin and the V-P model here. The pressure waveform imposed at the outlet boundary in the V-P model was the computational result obtained from the P-V model at the outlet. The matching of the blood pressure of the V-P model with the Pin at the inlet of the artery not only validates the numerical solutions of the V-P model, but also further confirms the validation of the numerical solutions of the P-V model.

## Results

**Figure 5:**
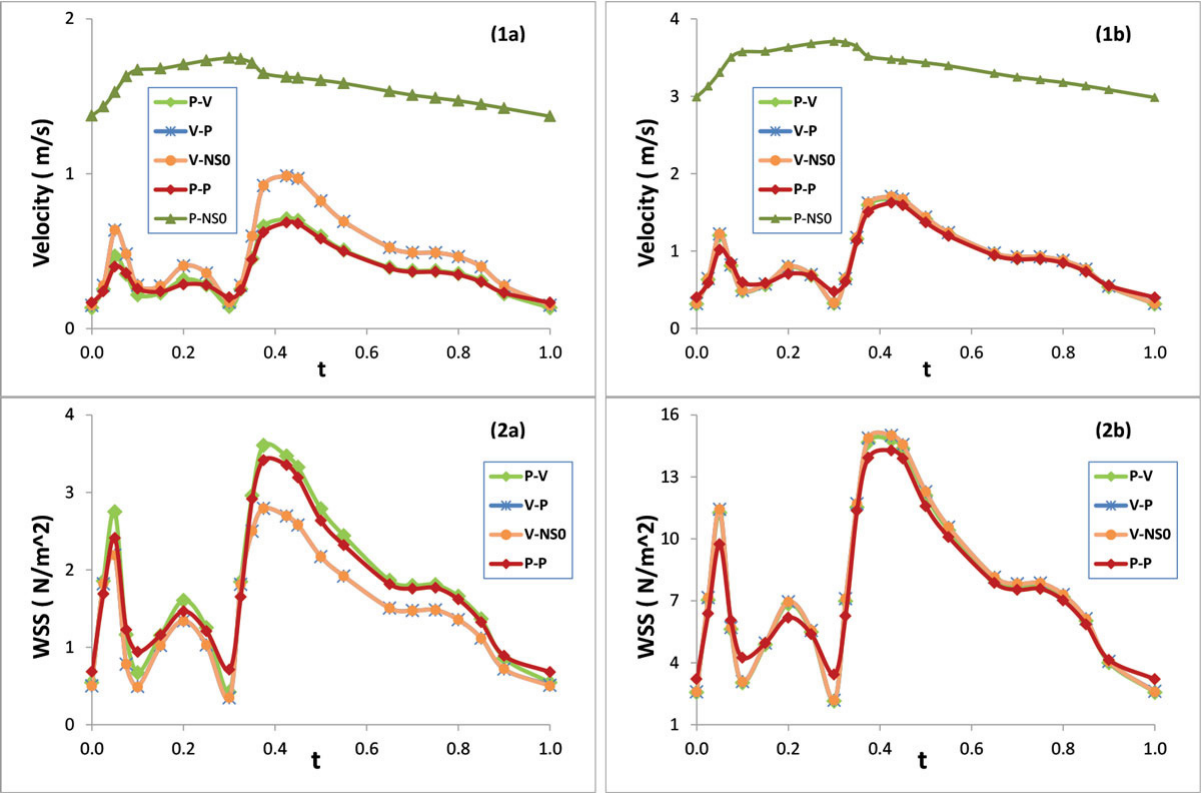
**Phasic plots of velocity magnitude at the center of the lumen cross-section at (1a) the inlet and (1b) the neck of the second stenosis; Phasic plots of the WSS at a point on the inner wall of (2a) the inlet and (2b) the neck of the second stenosis**.

**Figure 6:**
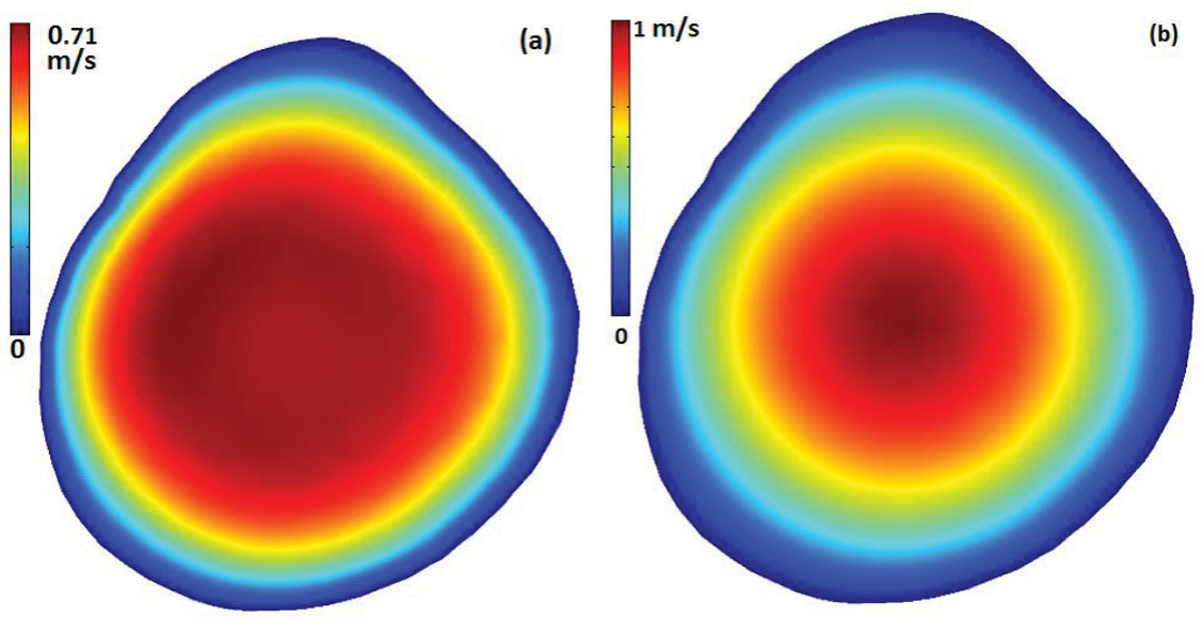
**Contour plots of the velocity magnitude at the inlet lumen cross section for (a) P-V/P-P models and (b) V-P/V-NS0 models**.

**Figure 7:**
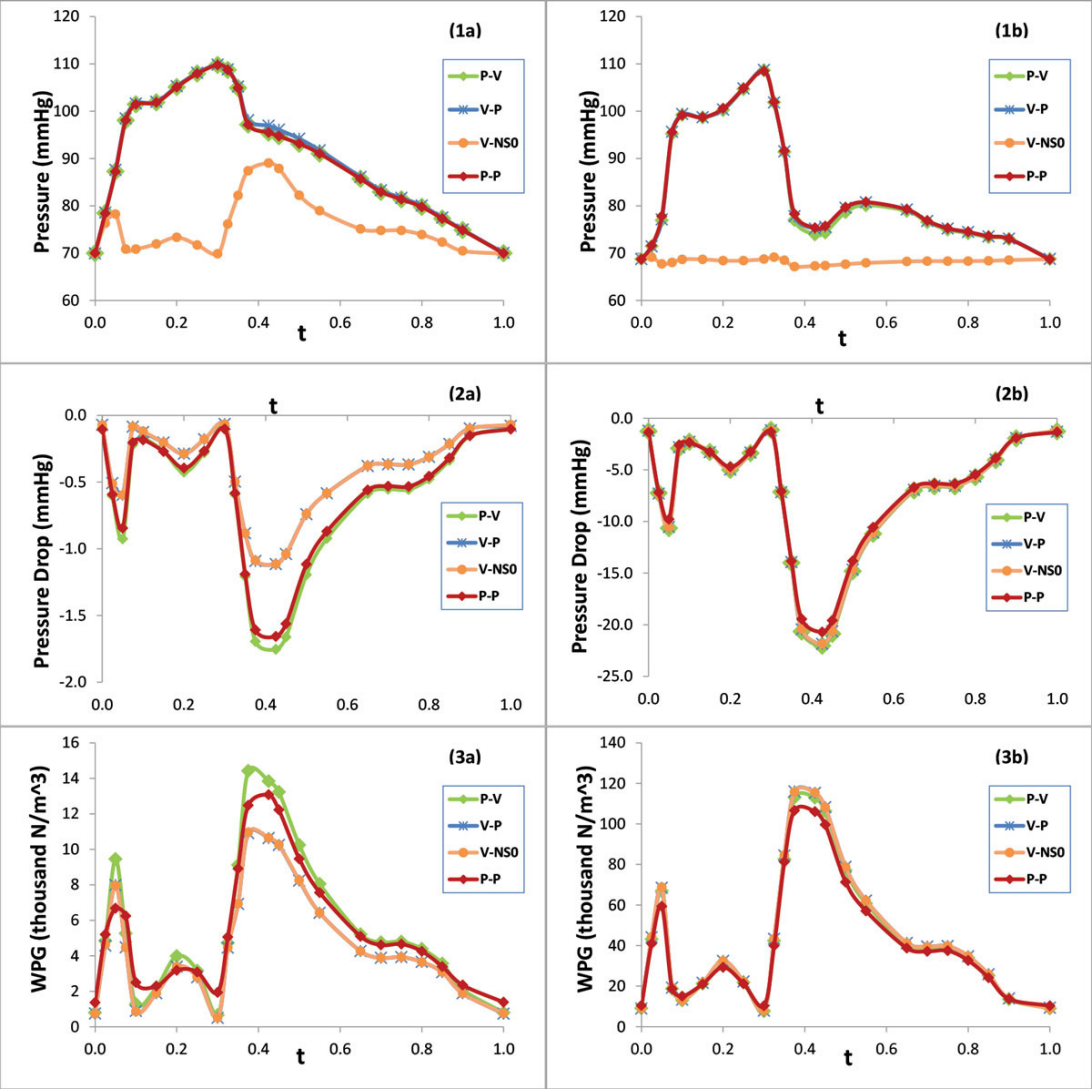
**Phasic plots of (1) the pressure, (2) the PD, and (3) the WPG at a point on the inner wall of (a) the inlet and (b) the neck of the second stenosis**.

**Figure 8:**
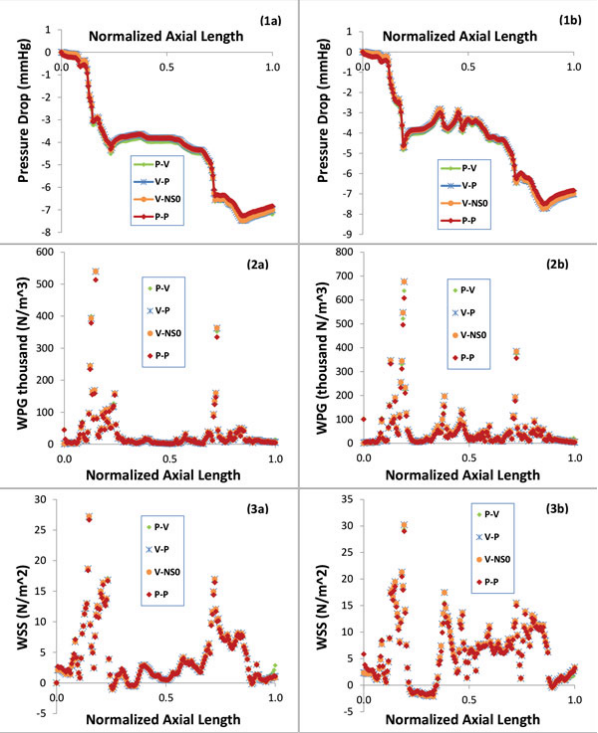
**Time averaged mean of (1) the PD, (2) the WPG, and (3) the WSS along (a) the inner curve and (b) the outer curve**.

Figure 5 presents the phasic plots of the magnitude of the velocity at the center of the lumen cross section at (1a) the inlet and (1b) the neck of the second stenosis for five models; Figure 5 also presents the wall shear stress at a point on the inner wall of (2a) the inlet and (2b) the neck of the second stenosis for P-V, V-P, V-NS0 and P-P models. Figure 6 includes the contour plots of the velocity magnitude at the inlet lumen cross section for (a) P-V/P-P models and (b) V-P/V-NS0 models. Figure 7 presents the phasic plots of (1) the pressure, (2) the pressure drop, and (3) the spatial gradient of the wall pressure at a point on the inner wall of (a) the inlet and (b) the neck of the second stenosis for P-V, V-P, V-NS0 and P-P models. Figure 8 shows the time averaged mean of (1) the pressure drop, (2) the spatial gradient of the wall pressure, and (3) the wall shear stress along (a) the inner curve and (b) the outer curve for P-V, V-P, V-NS0 and P-P models. Here the inner curve and the outer curve are the intersections of the axial cross section of the artery (x = 0) with lumen boundary on the inner border of the bend and on the outer border of the bend, respectively. The axial cross-section (x = 0) serves approximately as the middle-cut plane of the asymmetric artery. Table 1 lists the global maximum values of the mean PD, the mean WPG, and the mean WSS and the global minimum values of the mean WSS for five models. From the plots in Figures 5, 6, 7, 8, we can observe the effects of the boundary conditions (BCs) on the magnitude of the velocity, the WSS, the pressure, the PD, and the WPG. We can also see how the boundary conditions affect the temporal distribution patterns of the blood flows.

**Table 1:** Global maximums of the mean PD and the mean WPG, and the global maximum and minimum of the mean WSS for five models.

	V-NS0	V-P	P-V	P-P	P-NS0
Max PD_mean _(*mmHg*)	7.77	7.76	7.81	7.54	96

Max WPG_mean _(*N/m^3^*)	1470310	1474310	1415180	1360030	14690100

Max WSS_mean _(*N/m^2^*)	47.01	47.13	45.93	45.11	211.88

Min WSS_mean _(*N/m^2^*)	-7.84	-7.78	-7.61	-7.89	-61.43

### BCs have a notable effect on the velocity magnitude in upstream region

Figure 5(1a) indicates that the velocity magnitude of the P-V/P-P models is much lower than that of the V-P/V-NS0 models at the center of the inlet lumen cross section, while the velocity magnitude of the P-NS0 model is unrealistically high. The maximum velocity magnitude (0.708 *m/s*) in a cardiac cycle from P-V/P-P models was about 29% lower than that from V-P/V-NS0 models which used experimental velocity data. Both P-V and P-P models underestimate the blood velocity magnitude along the center line of the artery. With the existence of the secondary flow and the flow shifting in a stenotic curved artery, the blood velocity magnitude is highly non-uniformly distributed. The underestimate of the velocity magnitude of the P-V/P-P models on the center line varies significantly in different regions of the artery. Compared with the V-P/V-NS0 model, the velocity magnitude at the center of the 13^th ^lumen slice resulted from the P-V model or the P-P model is about 8% or 10% lower, respectively. These differences gradually diminish downstream along the artery.

The underestimation of the P-V/P-P models on the velocity magnitude at the center line in the upstream may be explained by the contour plots in Figure 6. All four models have the same flow rate during the cardiac cycle but with two different spatial distribution patterns of the velocity magnitude, one is blunter and the other is fully developed. The blunter inlet flow distribution of the P-V/P-P models results in a lower velocity magnitude at the center.

### Effect of the BCs on the WSS

Figure 5(2a&2b) show that the WSS at the inner wall resulted from different model is quite different, especially near the inlet of the artery. Compared with the V-P/V-NS0 models, the maximum wall shear stress at inlet in a cardiac cycle resulted from P-V model or P-P model is higher by 29% or 25%, respectively. The plots of the time average mean WSS along the inner curve and the outer curve in Figure 8(3a&3b) can further confirm the above differences observed. The V-P and the V-NS0 models underestimate the mean WSS by 8%, on average over the lumen wall of the upper stream segment, compared with the P-V model. There is no significant difference between the mean WSS of the P-P and the P-V models in this region (1.5% on average). The cause of the errors in this region maybe the assumption of the inlet fully developed spatial velocity profile on the V-P and the V-NS0 models. The blunter inlet flow distribution of the P-V/P-P models results in a higher WSS on the lumen wall than that of the V-P/V-NS0 models. As shown in Figure 5(2b) and Figure 8(3a&3b), the differences of the WSS between the V-P/V-NS0 and the P-V/P-P models decrease as flow moves from the inlet into the first stenosis. From the neck of the first stenosis to downstream, the WSS resulted from the V-P/V-NS0 models becomes slightly higher than that of the P-V/P-P models, with the maximum and the minimum mean WSS for all models listed in Table 1.

### BCs have notable influence on the pressure and the PD

Figure 7(2&3) and Figure 8(1)) indicate that the boundary conditions also have a non-negligible effect on the PD and the WPG, especially near the inlet. The V-P/V-NS0 models underestimate the pressure drop in upper stream segment of the artery. At the third lumen slice near the inlet of the artery, the pressure drop resulted from the V-P/V-NS0 models is less by about 36% at the peak flow (t = 0.425), compared with that resulted from the P-V model, which applied the *in vivo *data for both inlet and the outlet boundary conditions. This difference is gradually decreased till the neck of the first stenosis. The plots of the mean PD along the axial length of the artery in Figure 8(1a&1b) can further illustrate the above observations. Figure 7(3a&3b) show the similar pattern of the WPG on the difference between the V-P/V-NS0 models and the P-P/P-V models. Compared with the P-V model, the V-P/V-NS0 models underestimate the mean PD and the mean WPG by 43% and10%, respectively, on average over the lumen wall of the segment proximal to the first stenosis, while near the outlet of the artery, the V-P/V-NS0 models slightly overestimate the mean PD and the mean WPG.

### Effect of BCs on temporal distribution patterns of blood flows

The ways the boundary conditions are imposed impact the temporal distribution patterns of the blood flows. It is a popular approach in blood flow simulation that the outlet boundary condition is imposed with traction free/zero normal stress condition. When this outlet boundary condition is combined with a velocity or a pressure inlet boundary condition, the simulation may result in certain misleading temporal distribution patterns of blood flow, such as the pressure or the velocity.

As shown in Figure 7(1a&1b), the shape of the pressure phasic profile resulted from the V-NS0 model is very different from that of the pressure waveform extracted from the patient and the total variation of the pressure during a cardiac cycle is relatively small. The difference between the maximum and the minimum values of the pressure in a cycle is only approximately 20 mmHg at the inlet of the artery. The difference decreases along the axial length and the phasic waveform of the pressure becomes almost flat near the outlet of the boundary. It is obvious that the V-NS0 model can't provide correct information regarding the temporal distribution pattern of the pressure.

From Figure 5(1a&1b) we can see that the shape of the phasic profile of the velocity magnitude resulted from the P-NS0 model is not same as these of the other models. Instead, it is similar to that of the pressure waveform imposed at the inlet. The shape of the phasic profile of the pressure, the WPG, and the WSS for P-NS0 model are also all similar to that of the inlet pressure waveform. This is expected since the shape of the phasic blood flow profile follows the shape of the only pressure pulse waveform imposed at the boundary. The phasic velocity waveform of the P-NS0 model is clearly wrong compared with the velocity data extracted from the patient.

The shape of the phasic profiles of the PD, the WPG, the velocity, and the WSS for V-P model, P-P model, V-NS0 model, and P-V model are all similar to that of the velocity pulse waveform (Figure 5 and 7). This may suggest that when both the velocity and the pressure pulse waveforms are imposed at the inlet/outlet boundaries of the artery, the shape of the velocity pulse waveform dominates the temporal distribution patterns of both the velocity and the pressure difference related quantities, such as the PD, the WPG, velocity and the WSS. Even though there is no velocity waveform imposed directly on the boundaries for the P-P model, the reason for this observation still true is that the Pout applied at the outlet boundary was resulted from the computation of the P-V model, where the pulse waveform of Pout was already influenced by the velocity waveform.

### BCs have no significant effect on spatial distribution patterns of blood flows

**Figure 9:**
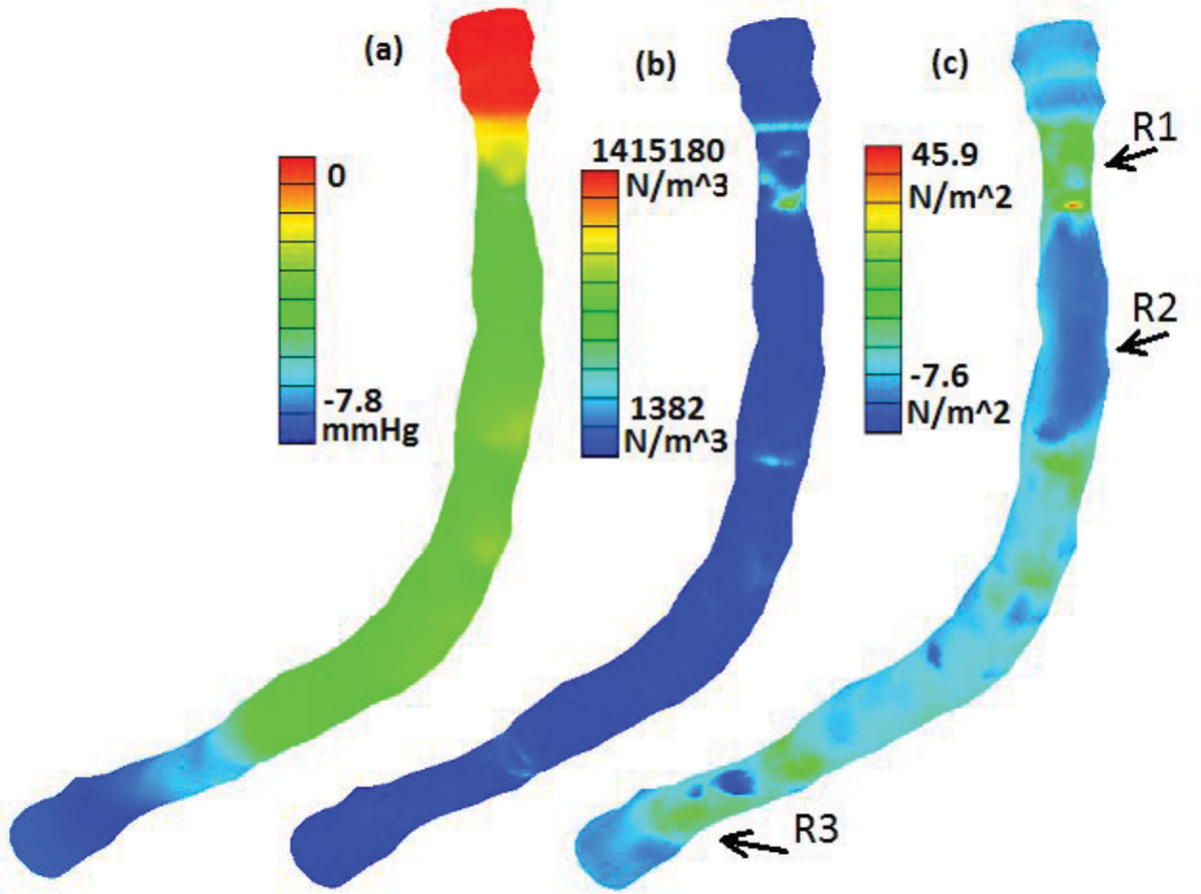
**Contour plots of (a) mean PD, (b) mean WPG, and (c) mean WSS for P-V model**.

The type of different boundary conditions imposed at the inlet and the outlet does not have significant effect on the spatial distribution patterns of the PD, the WPG and the WSS on the lumen surface of the artery, regarding the locations of the maximum and the minimum of each quantity. Figure 9 presents the contour plots showing the spatial distributions of the time averaged mean in a cardiac cycle of (a) the pressure drop, (b) the spatial gradient of the wall pressure, and (c) the wall shear stress along the artery lumen wall. In this plot, the artery was rotated by 45 degree about z-axis in order to have both the side-view and the outer border of bend-view of the artery. The plots included in this figure are the simulation results of the P-V model. The spatial distribution patterns of the mean PD, the mean WPG and the mean WSS obtained by the other four models are similar to those of the P-V model. Plots in Figure 9 demonstrate that the PD, the WPG and the WSS are highly non-uniformly distributed on the lumen wall of the atherosclerotic right coronary artery. We can see that around the neck of the first stenosis (labeled as R1 in Figure 9) the mean WPG and the mean WSS assume global maximum values and the wall pressure drops sharply. Another region with the high mean WPG and the high mean WSS is the neck of the second stenosis near the outlet (labeled as R3). Figure 9(c) also clearly show that the global minimum value of the mean WSS occurs in the region on the outer wall of post-stenosis (labeled as R2), where the lumen expands.

Plots in Figure 8 can further quantitatively confirm the observations on the spatial distribution patterns of the blood flow from Figure 9. The pressure has two sharper drops along the artery, each corresponding to the neck of a stenosis (see Figure 8(1a&1b)). These sharper drops of the wall pressure result in the elevated values of the spatial gradient of the wall pressure (see Figure 8(2a&2b)). The wall shear stress also peaks at the neck of each stenosis (see Figure 8(3a&3b)). Compared with the plots along the inner curve, the plots along the outer curve have some oscillations around the middle segment of the artery between the inlet and the outlet. It is probably due to the fact that the outer wall around the bend of the artery is less smooth. For the P-NS0 model (plots not included), the time averaged means of the pressure drop, the spatial wall pressure gradient, and the wall shear stress along the inner curve and along the outer curve have the similar patterns as those of other models, but with much larger values.

### When and where the WSS is less than 1 *N/m^2^*

Low and oscillating WSS have been associated with atherosclerotic lesions within the coronary arteries [21]. Intimal thickening likely occurs when the mean WSS is below 1 *N/m^2^*, which presents an inverse hyperplasia with respect to the shear stress [22]. It is of special interest to know the size of the region on the surface of the lumen where the WSS is lower than 1 *N/m^2 ^*and to examine the duration of the low WSS area in a cardiac cycle. At each point on the lumen surface, we calculated the total length of the time when the location experiences a low WSS < 1 *N*/*m^2 ^*in a cardiac cycle. We also recorded the distribution of the regions where the WSS is less than 1 *N*/*m^2 ^*at different time in the cardiac cycle and where the mean WSS is less than 1 *N*/*m^2^*.

**Figure 10:**
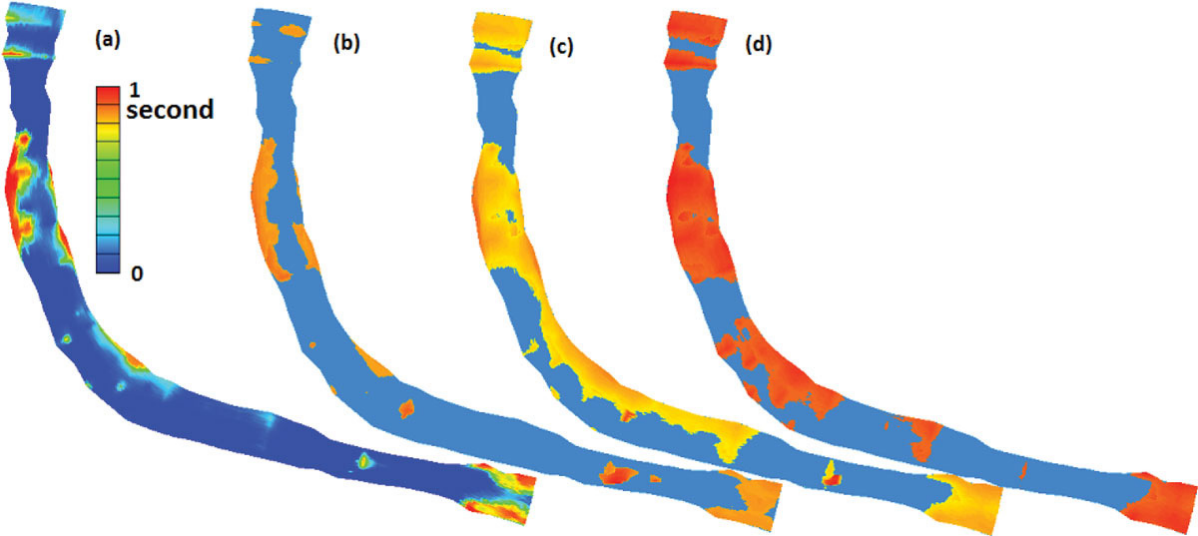
**(a) Contour plot of the total length of the periods in a cardiac cycle when the WSS is under 1 *N/m^2 ^*for V-P model**; (b) Areas where mean WSS < 1 *N/m^2 ^*for V-P model, (c) Areas where WSS < 1 *N/m^2 ^*when t = 0.3 for V-P model, (d) Areas where WSS < 1 *N/m^2 ^*when t = 0 for P-P model.

Figure 10(a) presents the contour plot of the total length of the time in a cardiac cycle when the WSS is under 1 *N/m^2 ^*for the V-P model. A point on the lumen surface with a value 1.0*s *as the total length of the time for the WSS under 1 *N/m^2 ^*indicates that the WSS at this location is lower than 1 *N/m^2 ^*anytime during the entire cardiac cycle. The legend for Figure 10(a) shows the variation of the cumulated time of the WSS under 1 *N/m^2 ^*at each point on the lumen surface. We can see that the post-stenosis regions of both stenoses experience the low WSS during the whole cardiac cycle. The orange-red liked color regions on the artery wall in Figure 10(b) represent the location and the size of the regions where the mean WSS of the V-P model is under 1 *N/m^2^*. Here the light blue was used as the base color for the artery wall. The pattern on Figure 10(b) is consistent with the result showing in Figure 10(a). The other four models have the similar spatial distribution patterns of the duration of the WSS under 1 *N/m^2 ^*as in Figure 10(a) and the patterns of the mean WSS under 1 *N/m^2 ^*as in Figure 10(b).

The size of each region with the WSS under 1 *N/m^2 ^*varies depending on the time stage in a cardiac cycle. The maximum size of the regions is assumed at certain time in a cycle depending on the type of boundary conditions of the model. For the models imposed with velocity profile boundary condition either at the inlet or at the outlet (V-NS0, V-P, P-V), the maximum size of regions with the WSS under 1 *N/m^2 ^*occurs at the minimum flow rate (t = 0.3) (see plots in Figure 10(c) for V-P model). For the P-P model, the maximum size of the regions with the WSS under 1 *N/m^2 ^*occurs at the beginning of the cardiac cycle (t = 0) (see Figure 10(d)). For the P-NS0 model, there is no significant difference for the size of regions with the WSS under 1 *N/m^2 ^*at different time during the cycle.

### Correlation of lumen cross-section area with the PD, the WPG, and the WSS

Table 2 presents the correlations of the lumen cross-section area with the slice averaged WSS_mean_, PD_mean_, and WPD_mean _for five models. Here WSS_mean_, PD_mean_, and WPD_mean _are the time averaged mean WSS, PD, and WPG in a cardiac cycle, respectively. The regression analysis used the lumen cross-sections of the 44 slice dataset. 128 mesh points were picked approximately evenly spaced on the lumen contour of each slice. The WSS_mean_, the PD_mean_, and the WPD_mean _were averaged on the picked mesh points of each slice, respectively, and regression analysis was performed on slice averaged base. As illustrated in Table 2 for all five models, a) the lumen cross-section area significantly correlates negatively with the time averaged mean of the wall shear stress and the spatial wall pressure gradient, and b) the lumen cross-section area correlates positively with the time averaged mean of the pressure drop. For example, the correlation coefficients of the lumen cross-section area with WSS_mean _and WPD_mean _are r = -0.79717 and r = -0.57274 with p-values as 0.0000, respectively. The correlation coefficient of the lumen cross-section area with PD_mean _is r = 0.47318 with a p-value of 0.0012 for V-NS0 model.

**Table 2:** Correlations of the lumen cross section area with the slice averaged WSSmean, PD_mean_, and WPG_mean_.

		V-NS0	V-P	P-P	P-V	P-NS0
WSS_mean_	r	-0.79717	-0.79715	-0.79055	-0.78983	-0.77805
	
	p	0.0000	0.0000	0.0000	0.0000	0.0000

PD_mean_	r	0.47318	0.47234	0.47253	0.47546	0.44946
	
	p	0.0012	0.0012	0.0012	0.0011	0.0022

WPG_mean_	r	-0.57274	-0.57289	-0.56663	-0.56255	-0.53660
	
	p	0.0000	0.0000	0.0000	0.0000	0.0002

## Discussion and conclusion

In literature, the effect of the inflow boundary condition was investigated by examining the impact of the waveform and the shape of the spatial profile of the inlet velocity on the cardiac hemodynamics. Waveform dependence has been cited for flow in the aortic arch [18,23] and in an end-to-side anastomosis [16]. The study conducted by Myers et al. implies that waveform shape is not a significant factor in the right coronary artery time-averaged WSS features. Changes in the inlet velocity profiles did not produce significant changes in the arterial velocity and wall shear stress patterns [1]. Our study was performed to examine the influence of the type of boundary conditions imposed at the inlet and the outlet on the phasic variation and the spatial distribution patterns of important right coronary artery hemodynamic features (velocity, the WSS, pressure, the PD, and the WPG) in a patient specific stenotic right coronary artery model.

Our results from this study indicate that the type of different boundary conditions imposed at the inlet and the outlet does not have significant effect on the spatial distribution patterns of the PD, the WPG and the WSS on the lumen surface of the artery in terms of where each quantity assumes maximum and where the minimum occurs. However, the temporal distribution patterns and the magnitudes of the velocity, the WSS, the pressure, the PD, and the WPG can be impacted notably depending on the type of the boundary conditions imposed at the inlet and the outlet. More in detail, it was observed that

• The type of boundary conditions examined in this study does not have a significant effect on the time averaged mean PD, the mean WPG and the mean WSS on the lumen surface in the post-stenosis and the downstream regions. However, the boundary conditions have notable effect in the segment of the artery between the inlet and the first stenosis on the magnitude of the velocity at the center line, on the pressure, the PD, the WPG, and the WSS. The fully developed velocity profile assumption maybe the cause of the difference. Therefore, if the local hemodynamics is the focus of the study, the inlet spatial velocity profile must be handled with caution for V-P and V-NS0 models.

• When the inlet boundary is imposed by a velocity waveform, the V-P and V-NS0 models with different outlet boundary conditions result in indistinguishable velocity, the PD, the WPG and the WSS. However, the temporal distribution patterns and the magnitudes of the pressure resulted from these two models are very different. The phasic variation of pressure in a cardiac cycle resulted from V-NS0 model is very different from that of the waveform extracted from the patient and thus may cause a misleading.

• When both of the velocity and the pressure waveforms are imposed at inlet/outlet boundaries, the shapes of the phasic profiles of the PD, the WPG, and the WSS only follow the shape of the velocity pulse waveform no matter whether it is imposed at the inlet or the outlet boundary. This may suggest that the velocity boundary condition influents more than the pressure boundary condition on the phasic shape of the PD, the WPG, and the WSS.

• P-NS0 model is not a practical mathematic model for the simulation of the blood flow in stenotic right coronary artery and it results in extremely large values of the PD and the WPD and a large variation of the WSS, which obviously are not realistic. It also results in a wrong shape of the pulse velocity waveform.

• The analysis of the sensitivity of the blood flow simulation on the pressure outlet boundary condition was also carried out. For the model with a pulse velocity waveform imposed at the inlet boundary (V-P), a 1% mean perturbation of the pulse pressure waveform (3% at the peak flow time) imposed at the outlet boundary will only cause a change of 0.5% in the maximum mean pressure drop and no change in the maximum mean WPG and the maximum mean WSS. On the other hand, for a model with a pulse pressure waveform imposed at the inlet boundary (P-P), the same amount of perturbation on the pulse pressure waveform imposed at the outlet boundary will result in a 7% change of the maximum mean PD, a 7% change of the maximum mean WPG, and a 5% change of the maximum mean WSS and a 6% change of the minimum mean WSS, respectively. This may suggest that the magnitudes of these hemodynamic features are more sensitive to the outlet pressure boundary condition in the P-P model than to that in the V-P model. Therefore, the accuracy of the measurement of the outlet pressure data from the patient is more important to the blood simulation in a patient specific P-P model.

• Comparing the plots of the inlet velocity and pressure waveforms with the plot of the pressure waveform at the outlet boundary, we can clearly see the influence of the flow rate on the pressure drop in a cardiac cycle. From the inlet to the outlet, the total pressure drop is larger at the time associated with each local maximum point of the inlet velocity waveform during a cardiac cycle, and the maximum pressure drop occurs at the peak flow (t = 0.425). A linear regression analysis on the correlation between the inlet pulse velocity magnitude and the percentage change of the pressure ((Pin(t)-Pout(t))/Pin(t)) reveals a correlation coefficient r = 0.9103 with p-value of 0.0000, and a linear fitting function: the percentage change of the pressure = 0.2134*velocity-0.0356 with R-square = 0.8286.

In conclusion, the observations based on the computer simulations in this study indicated that the ways how pressure and velocity boundary conditions are imposed in computational models have considerable impact on flow velocity and shear stress predictions, especially in upstream region. Computer simulations with improperly imposed type of boundary conditions may result in some misleading hemodynamic information, especially on the temporal distribution patterns and the magnitudes of the blood flow. Accuracy of *in vivo *measurements of blood pressure and velocity is of great importance for reliable model predictions. It must be recalled that we have examined only one artery in this work, and therefore any conclusions that we can draw are limited.

## Competing interests

Other than the grants listed in the acknowledgement section, the authors declare that they have no other competing interest.

## Authors' contributions

All authors actively contributed to the research and the writing of the manuscript. BL and DT contributed to the computational modeling, data analysis, and the draft of the manuscript. JZ and RB were responsible for data collection and image analysis, and active participation in manuscript writing. All authors 1) have made substantial contributions to conception and design, or acquisition of data, or analysis and interpretation of data; 2) have been involved in drafting the manuscript or revising it critically for important intellectual content; and 3) have given final approval of the version to be published. Each author has participated sufficiently in the work to take public responsibility for appropriate portions of the content.

## Declarations

Publication of this article was paid with funding from NIH/NIBIB R01 EB004759.

This article has been published as part of *BioMedical Engineering OnLine *Volume 14 Supplement 1, 2015: Cardiovascular Disease and Vulnerable Plaque Biomechanics. The full contents of the supplement are available online at http://www.biomedical-engineering-online.com/supplements/14/S1
